# Brain transcriptome variation among behaviorally distinct strains of zebrafish (*Danio rerio*)

**DOI:** 10.1186/1471-2164-13-323

**Published:** 2012-07-20

**Authors:** Robert E Drew, Matthew L Settles, Erin J Churchill, Shayna M Williams, Soniya Balli, Barrie D Robison

**Affiliations:** 1 Department of Biological Sciences and Program in Bioinformatics and Computational Biology, University of Idaho, Moscow, ID, 83844, USA; 2 Department of Biology and Program in Biomedical Engineering and Biotechnology, University of Massachusetts—Dartmouth, North Dartmouth, MA, 02747, USA; 3 Department of Computer Science and Program in Bioinformatics and Computational Biology, University of Idaho, Moscow, ID, 83844, USA

**Keywords:** Zebrafish, Transcriptome, Microarray, Behavior, Anxiety, Domestication

## Abstract

**Background:**

Domesticated animal populations often show profound reductions in predator avoidance and fear-related behavior compared to wild populations. These reductions are remarkably consistent and have been observed in a diverse array of taxa including fish, birds, and mammals. Experiments conducted in common environments indicate that these behavioral differences have a genetic basis. In this study, we quantified differences in fear-related behavior between wild and domesticated zebrafish strains and used microarray analysis to identify genes that may be associated with this variation.

**Results:**

Compared to wild zebrafish, domesticated zebrafish spent more time near the water surface and were more likely to occupy the front of the aquarium nearest a human observer. Microarray analysis of the brain transcriptome identified high levels of population variation in gene expression, with 1,749 genes significantly differentially expressed among populations. Genes that varied among populations belonged to functional categories that included DNA repair, DNA photolyase activity, response to light stimulus, neuron development and axon guidance, cell death, iron-binding, chromatin reorganization, and homeobox genes. Comparatively fewer genes (112) differed between domesticated and wild strains with notable genes including *gpr177* (*wntless*), selenoprotein P1a, synaptophysin and synaptoporin, and acyl-CoA binding domain containing proteins (*acbd3* and *acbd4*).

**Conclusions:**

Microarray analysis identified a large number of genes that differed among zebrafish populations and may underlie behavioral domestication. Comparisons with similar microarray studies of domestication in rainbow trout and canids identified sixteen evolutionarily or functionally related genes that may represent components of shared molecular mechanisms underlying convergent behavioral evolution during vertebrate domestication. However, this conclusion must be tempered by limitations associated with comparisons among microarray studies and the low level of population-level replication inherent to these studies.

## Background

The process of animal domestication is a striking example of convergent behavioral evolution. During domestication, populations diverge from their wild progenitors as a result of genetic drift, artificial selection on desirable traits, relaxed selection on previously important characteristics, and adaptation to new environmental and ecological conditions [1-3]. Evolution in captivity is associated with a set of phenotypic changes, including enhanced growth rate [4-6], attenuated responsiveness to stress [7,8], increased feeding behavior [9], and reduced predator avoidance behavior [6,10,11]. Reductions in predator avoidance behavior are the most consistent responses to domestication and have been observed across a broad range of taxa, including mammals, birds, and fish [12-14]. However, it is not known whether the apparent convergent evolution of predator avoidance behavior during domestication is the result of similar genetic changes or of changes in a myriad of alternative molecular “routes” to the same phenotypic endpoint. In fact, little is known about the genetic polymorphisms associated with domestication in any vertebrate species.

Differences in gene regulation are an important source of genetic variation for phenotypic evolution [15-17]. Transcriptome analyses have detected substantial variation among populations in numerous species, including yeast [18], *Drosophila*[19], fish [17,20-24], and humans [25,26]. Much of this variation has a heritable component [25,27-30]. While the majority of variation in gene expression appears to evolve in a neutral fashion, some genes show patterns consistent with evolution under positive selection and may contribute to local adaptation [17,29,31-33], including behavioral adaptations [20,34]. In particular, microarray studies suggest that domestication may also be associated with changes in gene expression [22,35-37].

In the present study, we use the zebrafish (*Danio rerio*) as a model for examining the relationship between behavioral domestication and variation in gene expression. The utility of the zebrafish in developmental biology and genomics is widely known, and there has been increased usage of the zebrafish as a model for behavior genetics [38-41]. Variation in behavior has been observed among populations of zebrafish, including variation among domesticated (laboratory) populations [38], among populations recently acquired from the wild [42], and between wild and domesticated populations [9,43-45]. In each of these studies, the populations were reared under identical conditions suggesting that the differences in behavior have a genetic component [9,43,44].

In our study, we used microarrays to perform a transcriptome-wide analysis of variation in brain gene expression among four behaviorally distinct strains of zebrafish with different histories of domestication. First, we quantified behavioral differences between wild and domesticated zebrafish strains using a high throughput behavior assay [46]. Second, using microarrays, we detected substantial variation in expression profiles among these behaviorally distinct populations, a portion of which was associated with domestication history. Finally we compared our results against other microarray studies [22,35-37,47] and identified sixteen homologous genes that were associated with behavioral domestication and variation in fear-related behavior across multiple species.

## Results and discussion

### Behavioral variation among zebrafish strains

We used a simple, high-throughput assay to characterize behavioral differences among zebrafish strains [46]. The assay was based on initial observations that fish from domesticated strains tended to spend more time near the surface and at the front of the aquarium nearest human technicians while wild fish tended to be found at the back and near the bottom of the aquarium. This assay decomposes this place preference type behavior into a vertical component (Vertical Depth) and a horizontal component (Horizontal Position). Vertical Depth quantifies the mean distance of an individual from the water surface while Horizontal Position is the proportion of the time an individual spends within one body length of the front of the tank, nearest to a human observer.

Although simple in design, the behaviors quantified by this assay can be used as indices of fear-related behavior in fish species, and are likely indicators of trade-offs between foraging and predator avoidance. In the wild, proximity to the surface is believed to increase the risk of predation by aerial predators [13,48,49] and observations of zebrafish and other fish species are consistent with this hypothesis [50,51]. However, in captivity, fish are commonly offered food that floats on the surface, providing a contradictory stimulus for approaching the surface. Indeed, mean distance from the surface is negatively correlated with feeding behavior in zebrafish (M. Oswald and B.D.R., Unpublished data), masu salmon (*O. masou*) [52], and growth hormone transgenic coho salmon (*O. kisutch*) [53]. Also distance from the surface is positively correlated with freezing in response to a simulated predator by masu salmon [52]. Recent work has also shown that zebrafish spend less time in the upper half of aquaria in response to stressful stimuli and treatment with anxiogenic chemicals (alarm pheromone and caffeine), and more time following treatment with anxiolytic compounds (ethanol and fluoxetine) [54]. Therefore we conclude that these behaviors are valid indicators of fearfulness in various fish species.

**Figure 1:**
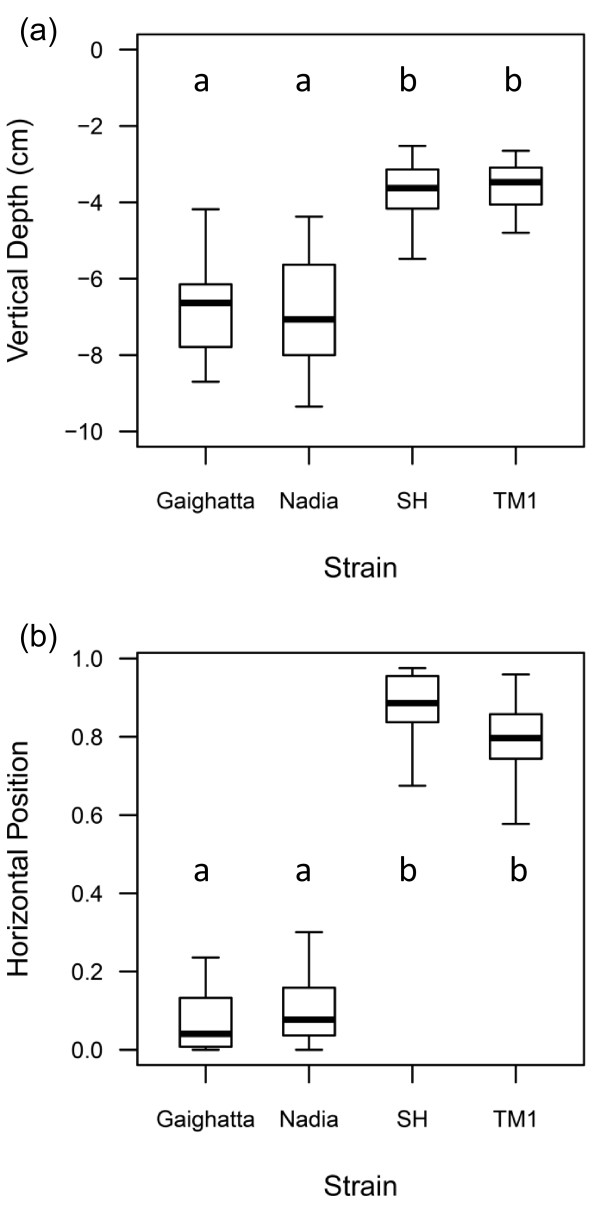
**Box plots of behavioral variation among two wild and two domesticated zebrafish strains.** (**a**) Domesticated zebrafish [Scientific Hatcheries (SH) and TM1] had lower Vertical Depth, spending more time close to the water surface than wild zebrafish [Nadia and Gaighatta] (*P* < 0.0001). (**b**) Domesticated zebrafish also had higher levels of Horizontal Position, spending a larger portion of time within one body length of the front of the aquarium nearest the human observer (*P* < 0.0001). Boxes represent the 25^th^ and 75^th^ percentiles, while the median is indicated by the interior horizontal line. The 5^th^ and 95^th^ percentiles are represented by the whiskers. Significant differences among strains are represented by lower case letters within each figure.

We used these behavior assays to characterize four strains of zebrafish with different histories of domestication. The Scientific Hatcheries and Transgenic Mosaic 1 (TM1) strains have been reared in the laboratory for at least 30 generations and will be referred to as “domesticated.” The Gaighata and Nadia strains were recently acquired from wild populations in India and, at the time of this study, had been reared in captivity for 1 and 6 generations, respectively. For the sake of brevity, these strains will be referred to as “wild.” Although all of the fish used in this experiment were bred and reared under identical captive conditions (“common garden”), there were significant behavioral differences between wild and domesticated zebrafish strains. The wild strains spent markedly less time within one body length of the front of the aquarium, nearest the observer (low Horizontal Position, F = 208.45, df = 3, 58, *P* < 0.0001), and showed a greater preference for the lower portions of the water column (greater Vertical Depth, F = 25.46, df = 3, 58, *P* < 0.0001) than the domesticated strains (Figure 1).

The differences in behavior between wild and domesticated zebrafish populations were consistent with previous observations of these and other strains of zebrafish [9,43-45]. In particular, these four strains vary in their latency to feed from the surface of the water such that wild fish take much longer to commence feeding than domesticated fish [9]. Likewise, similar differences have been observed between wild and domesticated populations of other fish species (primarily salmonids) [13,48,55-59]. In general, domesticated fish tend to be bolder, feed more frequently, and have reduced ability to avoid predation [13], characteristics that have been linked with decreased survival in a wild environment [60].

### Transcriptome variation among zebrafish strains

We next tested if these behaviorally distinct zebrafish strains also differed in gene expression in the brain to identify genes or pathways that may underlie behavioral variation. GeneChip® Zebrafish Genome microarrays (Affymetrix, Santa Clara, CA) were used to quantify the expression of approximately 14,900 zebrafish transcripts in the brains of fish observed in the behavior study above.

A total of 7,958 probe sets were detected as expressed in our zebrafish brain samples, based on our filtering criteria for expression (see Methods). Of these, 3,199 probe sets displayed probe level variation indicative of sequence polymorphisms among individuals and populations, known as single feature polymorphisms (SFPs) [61-63]. The presence of SFPs can confound comparisons of gene expression among different genotypes and therefore these features were removed from the analysis.

The remaining 4,759 probe sets were analyzed using linear models for microarrays (limma) [64,65] to test for differences in transcriptome profiles among strains, between wild and domesticated strains, and between males and females. Our initial analysis revealed considerable variation in gene expression among the strains with 1,752 probe sets differentially expressed among strains, 259 with a 2-fold or greater difference in pairwise comparisons of mean expression levels [see Additional file 1. This level of brain transcriptome variation among populations is substantially higher that than observed between two populations of rainbow trout (201 genes, [37]) and among eight behaviorally diverse inbred mouse lines (188 genes, [47]); however these differences may reflect differences in statistical analyses or gene content of the array platforms.

The three probe sets with the highest differences among strains corresponded to quality control probe sets for green fluorescent protein (GFP: AFFX-Dr-M62653-1_at, AFFX-Dr-M62653-1_s_at) and cyan fluorescent protein (CFP: AFFX-Dr-AF292560-1_s_at). Because the expression of GFP in the TM1 strain was the result of transgenic manipulation [66], these genes were removed from the analysis. The next ten most differentially expressed zebrafish genes included cytochrome P450, family 1, subfamily A (*cyp1a*); major histocompatibility complex class II DAB gene (*mhc2dab*); G protein-coupled receptor 177 (*gpr177*); and seven unannotated sequences.

The Database for Annotation, Visualization and Integrated Discovery (DAVID version 6.7) [67,68] was used to identify functional categories that were overrepresented in the final gene lists (excluding SFPs) relative to the 7,958 genes detected as expressed in our analysis. DAVID identified 116 functional categories that were significantly overrepresented among genes that varied in expression among strains [see Additional file 2. Notable functional categories included those involved in DNA repair, DNA photolyase activity, response to light stimulus, neuron development and axon guidance, cell death, iron-binding, WD40 repeat domains, chromatin organization, and homeobox genes. Functional interpretation of these findings is complicated because, although these genes differed among zebrafish populations, variation in expression was distributed in different patterns among the four zebrafish populations and did not necessarily involve correlated expression. These categories may represent functional groups of genes that, in the brain, may frequently vary among zebrafish populations. It is not known if this variation in gene expression is under selection or whether it is associated with variation in brain function or behavior.

### Transcriptome variation between wild and domesticated strains

A total of 612 probe sets were initially identified as differentially expressed between wild and domesticated strains. Visual inspection of the data suggested that many of these differences were driven by extreme values in one of the strains. To increase the stringency of the analysis, genes associated with domestication were retained if the ranges of expression levels of wild and domesticated samples overlapped by no more than one sample. This additional criterion narrowed the list to 112 genes that were differentially expressed between wild and domesticated samples, 10 of which had a 2-fold difference or greater [see Additional file 3].

G protein-coupled receptor 177 (*gpr177*) showed the largest difference with a 5.59-fold down-regulation in domesticated zebrafish. *gpr177*, also known as *wntless* or evenness interrupted (*evi*), is necessary for the release of the WNT signaling peptide from WNT-secreting cells [69,70]. The WNT signaling pathway is a major control pathway for many aspects of embryonic development, including patterning of the central nervous system, and is also involved in axon guidance and synapse formation in adult organisms [71,72]. In addition, defects in WNT signaling are associated with neurological disorders in humans, including schizophrenia and Alzheimer’s disease [73]. Selenoprotein P (*sepp1a*) was also among the most differentially expressed genes with a 1.85-fold up-regulation in domesticated zebrafish, similar to previous observations of these strains [43]. The role of SEPP in the brain is not completely understood but the protein plays a vital role in selenium homeostasis [74]. Mouse SEPP knockout mutants have reduced ability to store selenium in the brain and other tissues, and show severe neurological impairment [75-77]. In humans, selenium levels are associated with disease states including epilepsy, Parkinson’s disease, and Alzheimer’s disease [74]. Although it is unknown if SEPP plays a role in these disorders, sequence variation in *sepp* affects the ability to utilize selenium in humans [78].

DAVID identified seven functional categories that were overrepresented among all genes differentially expressed between wild and domesticated zebrafish. After accounting for overlap, the functional groups can be reduced to two categories: vesicle proteins and acyl-CoA binding domain containing proteins. Each of these groups was represented by at most two or three individual genes. The vesicle proteins included synaptophysin b and synaptoporin which are associated with synaptic vesicles and synaptic plasticity [79,80], and SEC31 homolog, a subunit of the coat protein complex II which enables the formation of transport vesicles from the endoplasmic reticulum. Knockout mice lacking synaptophysin are viable but show increased exploratory behavior and reduced learning ability and memory [81], indicating that this gene can be directly involved in behavioral variation. Stress is also known to influence both mRNA and protein levels of synaptophysin in rats but the relationship is complex and influenced by factors including sex and the severity of the stressor [82-84]. Reduced sensitivity to stress has been observed in domesticated fish [8,85] and may be a factor in our study. The acyl-CoA binding domain containing proteins included *acbd3* and *acbd4*. ACBD3 maintains the structure of the Golgi apparatus, influences protein transport between the Golgi apparatus and the endoplasmic reticulum, and is an important component of several cellular signaling pathways (reviewed by [86]). The function of ACBD4 has not been determined.

### Transcriptome variation between zebrafish sexes

Although not a primary focus of this experiment, we also tested for sex differences in gene expression. We detected only eight sexually dimorphic genes in the brain: insulin-like growth factor 1 (*igf1*); deiodinase, iodothyronine, type II (*dio2*); inhibin, beta Aa (*inhbaa*); endothelial PAS domain protein 1 (*epas1*); and four unannotated genes (Dr.14275.1.A1_at, Dr.16113.1.S1_at, Dr.16580.1.A1_at, Dr.18139.1.S1_at, Dr.18392.1.A1_at, and Dr.6751.1.S1_at). Of these only *dio2* was previously documented as sexually dimorphic in zebrafish brains [43,87]. Interestingly, although 40 of the 42 sexually dimorphic genes identified by Santos *et al.*[87]were represented on the GeneChip® Zebrafish Genome microarray, only eleven were detected as expressed in our study. This lack of concordance with our study is surprising, but could result from differences in experimental procedures, microarray platform and chemistry, and statistical analysis. A higher number of sexually dimorphic genes (81 genes) were also detected in brain transcriptomes of immature Atlantic salmon (*Salmo salar*) [34]. Despite the differences between these studies and ours, all three studies showed substantially fewer sexually dimorphic genes in the brain relative to other tissues, such as liver (1249 genes [88]) and gonads (2940 genes [89]). This result is comparable to observations in mammals [90].

### Validation using quantitative real-time PCR

**Figure 2:**
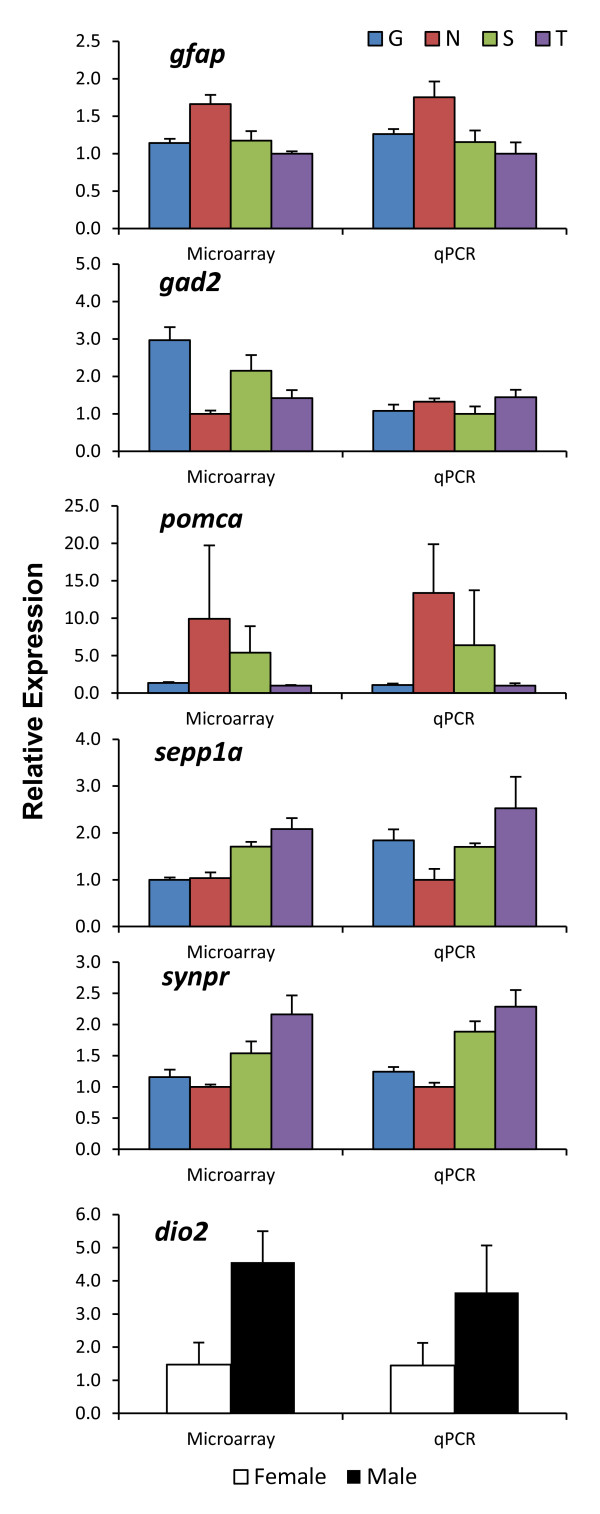
**qRT-PCR was used to validate microarray results for six genes.** Microarray and qRT-PCR showed similar patterns for five of the six genes: glial fibrillary acidic protein (*gfap*), proopiomelanocortin a (*pomca*), selenoprotein P1a (*sepp1a*), synaptoporin (*synpr*), and deiodinase, iodothyronine, type II (*dio2*). Validation failed for glutamate decarboxylase 2 (*gad2*), suggesting that additional factors may be affecting hybridization to the microarray. Abbreviations: G = Gaighatta, N = Nadia, S = Scientific Hatcheries, T = TM1.

We performed a technical validation using quantitative real-time PCR (qRT-PCR) to verify expression of six differentially expressed genes identified by the microarray analysis. The genes selected for qRT-PCR validation differed significantly among strains (*gad2gfappomca*), between wild and domesticated strains (*sepp1asynpr*), or between the sexes (*dio2*) in the microarray analysis. The qRT-PCR results were consistent with the microarray analysis for four of the six genes (Figure 2). qRT-PCR measures of *gad2* expression did not agree with the microarray results, suggesting that there may be additional factors affecting hybridization to the microarray. Expression of *sepp1a* also differed between microarray and qRT-PCR in that the Gaighatta strain had higher levels than expected; however, we previously found agreement between our microarray findings (current study) and qRT-PCR measures of *sepp1a* in an independent sample of these strains [43]. This unexpected disagreement may reflect strain-specific regulation of *sepp1a* expression by dietary selenium levels [43].

### Comparison with other studies

Examination of these findings in a comparative context can allow us to prioritize genes for future analyses. We therefore compared our results to other analyses in zebrafish and other species. Several microarray studies have examined transcriptome variation associated with domestication or fear-related behavioral variation in other vertebrates. These studies include a comparison between wild and domesticated rainbow trout [37], an analysis of the effect of selection for tame behavior in gray fox [35], a comparison between dogs and wolves [36], and an analysis of behavioral variation among inbred mouse lines [47]. Cross-species comparisons of microarray studies are complicated by differences in microarray platforms and chemistry, hybridization efficiencies, experimental design, and statistical analyses. The studies also differed at the scale at which gene expression was measured in the brain. The canid and mouse studies measured expression separately in multiple regions of the brain [35,36,47] while our study and the rainbow trout study [37] examined gene expression in the entire brain. Regional analyses of gene expression may be able to detect local changes in gene expression that are not detectable in whole brain analyses [91] but brain regions must be selected with care to ensure that relevant regions are included in the analysis.

Despite these complications, we identified sixteen evolutionarily or functionally related groups of genes that were associated with domestication in five vertebrate species, or fear-related behaviors in mice (Table 1). The association of these genes with independently derived domesticated populations of evolutionarily divergent species suggests that these genes may be components of pathways influenced by convergent evolution during domestication, although they may not represent the causative polymorphisms. These genes are involved in a variety of functions including translation initiation, protein folding (DNAj genes), amelioration of oxidative stress (glutathione peroxidases and selenoprotein P), and metabolic function (cytochrome c oxidases, NADH dehydrogenases). However, a few are known to affect functions in the brain.

**Table 1:** Related genes with brain expression patterns associated with domestication in multiple species

**Genes**	**Zebrafish Strain**	**Zebrafish Domestication**	**Rainbow Trout Domestication[**[37]**]**	**Fox Tameness[**[35]**]**	**Dog-Wolf Comparison[**[36]**]**	**Mouse Behavior[**[47]**]**
Caspase	casp2, casp3a, caspb	casp3a (+)	casp8 (+)			
Coiled-coil domain containing	ccdc47, ccdc53, ccdc93, Dr.18793.1.A1_at	Dr.18793.1.A1_at (+)	BU965755 (+)			
Collagens	col1a2, col2a1a, col5a2l, col9a2, wu:fa99c11, zgc:56518		col1a1 (+), col1a2 (+)			col1a1^a^, col3a1^b^
Cytochrome c oxidase			cox2 (–), cox3 (–)			cox5a^b^
DNAj	dnajc12	dnajc3 (–)				dnajc6(AI840916)^c^
Eukaryotic translation initiation factors	eif1ax, eif4enif1	eif1ax (+), eif4enif1 (–)	eif3s2 (+), eif3s7 (+)			
Glutathione peroxidases	gpx1a, gpx4a		gpx3 (–)			gpx3^d^
Hemoglobins	hbaa1, ba1		hba,hba1, hba4, hbb, hbb1, hbb2, hbb4 (all +)	hbe, hbg1, hbg2, hba, hbz (all –)		hbaa1^e^
NADH dehydrogenase (ubiquinone)			ndf1a4 (+)	ndufb8 (–)		
Neurofilament, light peptide			CB514125 (+)			nefl^e^
Potassium channel proteins	Dr.16785.1.A1_at (kcnk1)				kcnj4 (–)	AI835316 (kcnc2)^a^
Purkinje cell protein 4				pcp4 (*)	pcp4 (+)	
Selenoprotein P	sepp1a	sepp1a (+)			sepp1 (+)	
Syntaxins	stx3a, stx6, syntaxin1b			stx1b2 (+)		
Syntaxin binding proteins			stxbp3 (+)			stxbp2^b^, stxbp6^e^
Transthyretin (prealbumin, amyloidosis type I)				ttr (*)	ttr (+)	

Studies included the present study on zebrafish (*Danio rerio*), an analysis of domestication in rainbow trout (*Oncorhynchus mykiss*) [37], silver fox (*Vulpes vulpes*) selected for tameness [35], a comparison between dog (*Canis familiaris*) and wolf (*Canius lupus*) [36], and an analysis of strain variation in laboratory mice (*Mus musculus*) [47]. Positive signs (+) indicated up-regulation in domesticated populations relative to wild, while negative signs (–) indicate down-regulation. An asterisk (*) indicates that the direction of differential expression differed among brain regions.

^a^ Negative correlation with open field activity; ^b^ Positive correlation with distance traveled after ethanol administration; ^c^ Positive correlation with electroconvulsive threshold; ^d^ Negative correlation with electroconvulsive threshold; ^e^ Positive correlation with open field activity.

A few of these genes are associated with synapse function and long-term potentiation. Syntaxins are components of the SNARE complex which mediates the docking of vesicles with extracellular membranes, including the release of neurotransmitters from axon terminals [92], and appears to be regulated by several syntaxin-binding proteins [93,94]. Protein levels of syntaxins increase during long-term potentiation in the rat hippocampus [95]. In studies related to domestication, mRNA expression of *syntaxin1b2* was higher in foxes selected for tameness [35]; expression of syntaxin-binding proteins was higher in domesticated rainbow trout [37] and correlated with fear-related behaviors in mice [47]. In contrast, in our study of zebrafish, expression of three syntaxin genes differed among strains but was not associated with domestication history. Likewise, caspases, such as capase-3, are associated with synaptic plasticity and learning, in addition to their involvement in apoptosis [96,97]. *Caspase-3a* and *capase-8* were up-regulated in domesticated populations of zebrafish (current study) and rainbow trout [37]. These observations suggest that synapse development and function may be influenced by domestication.

The association of collagens and hemoglobins with domestication of multiple species was surprising. Collagens are rare in the brain and are usually associated with the meninges surrounding the brain [98]. This includes several of the collagen genes differentially expressed among zebrafish populations (present study), between wild and domesticated rainbow trout [37], and correlated with behavior in mice [47]. It is possible that the meninges may have contaminated the brain samples, although it is not clear why this contamination would differ in level between populations in multiple species. Alternatively a few collagen genes are expressed in the brain and appear to be involved in neurological development, including axon guidance and synaptogenesis in the CNS [98,99].

Expression of hemoglobin genes was positively associated with domestication in rainbow trout [37], negatively associated with tameness in foxes [35], and positively associated with open field activity in mice [47]. Hemoglobin genes were also differentially expressed among zebrafish populations. It was recently discovered that hemoglobin genes are expressed in neurons, astrocytes, and oligodendrocytes of rats and humans, and may be involved in oxygen storage and mitochondrial function in the brain [100,101].

Finally, SNP variation has been detected among zebrafish populations and may be associated with the variation in behavior and gene expression we observed here. Whiteley *et al.*[102] examined SNP variation among thirteen wild populations and three laboratory strains of zebrafish and detected 99 significantly divergent outlier SNP loci that may be signatures of natural selection. Eight of these outlier loci occurred in genes that were also differentially expressed in our analysis among populations (*arg2gpx1aphopspc1*, si:dkey-15j16.2, and zgc:77304) and between wild and domesticated populations (*bhlhe40* and *syntaxin1b*), suggesting that variation in the expression of these genes may also be under selection. Their study populations included the laboratory TM1 strain and wild populations in geographic regions from which our wild strains were originally collected.

## Conclusion

Using a high throughput behavior assay, we detected significant variation among zebrafish strains in behaviors related to fearfulness and predator avoidance. This variation showed a strong association with domestication history, consistent with patterns observed in other fish species. While there was substantial inter-strain variation in patterns of gene expression in the brain, far fewer genes were associated with domestication history. This suggests two hypotheses regarding the evolution of behavior during domestication. First, convergent behavioral evolution during domestication may result through independent alterations in different pathways that achieve similar phenotypic effects. If this is the case, each of our domesticated zebrafish populations would have different genes associated with behavioral variation, and these genes would be lost among the large number of genes varying among populations and therefore undetectable in our comparison. Alternatively, evolution of these behavioral phenotypes may involve only a few genes or pathways to achieve the behavioral variations observed here. In our study, the number of genes associated with domestication in zebrafish is comparable to that associated with within-population behavioral variation in other fish species, such as differences between dominant and subordinate cichlids (*Astatotilapia burtoni*, 171 genes, [103]) and between sneaker and migratory males in Atlantic salmon (*Salmo salar*, 432 genes, [34]). This suggests that behavioral variation can be associated with alterations in the expression of relatively few genes in the brain. In addition, identification of genes associated with domestication in multiple vertebrate species may indicate convergent evolution of a few key molecular pathways during domestication. Full evaluation of these hypotheses is beyond the scope of our study and would require more comprehensive analyses using both multiple populations and multiple species. However transcriptome variation among populations can be substantial and should be given careful consideration when designing genomic analyses of behavior and other traits.

## Methods

### Zebrafish strains

We used zebrafish from four distinct strains for our experiments. Two of these strains have been reared in captivity for more than thirty generations each [9,43,44]. The Scientific Hatcheries strain (SH) was originally obtained from a commercial breeder (Scientific Hatcheries, Huntington, CA) and reared in our laboratory for three generations prior to the experiment. It is difficult to determine the total number of generations for which this strain has been in captivity, but it is known to be at least thirty. The TM1 strain was founded from a pet store population and reared for 24 generations at the University of Miami [66] and a further six generations prior to the experiment at the University of Idaho. The TM1 strain is GFP transgenic using a β-actin promoter [66]. Two additional strains, Nadia and Gaighata, were recently derived from wild populations in India. The Nadia strain was originally collected in India in 1999. At the time of this study, the strain had been reared in the laboratory for six generations, remaining qualitatively similar to the original wild collections [9,43,44]. The Gaighatta strain was collected in India in 2005, and was in its first generation of lab rearing at the time of this study.

Prior to the experiment, all zebrafish were reared in a recirculating zebrafish facility at the University of Idaho, designed by Aquaneering Inc. (San Diego, CA). Fish were feed twice daily with a combination of commercial flake food and *Artemia* nauplii. Temperature of the facility was maintained at 28 °C with a constant 14 h light: 10 h dark cycle. These rearing conditions were maintained during the behavior assays. All procedures involving animals were approved by the University of Idaho Animal Care and Use Committee.

### Behavior assays

Sixteen adult fish (4 to 6 months old) from each strain were randomly assigned to 1-L aquaria (22 cm long, 15 cm high, 5 cm wide), one fish per aquarium. The aquaria were delimited into six 2-cm vertical zones using thin twine tied across the front of the entire row of aquaria. Fish were visually isolated by placing paper barriers between aquaria. Each aquarium was labeled with a unique numerical identifier so that information regarding strain and sex was unknown to the observer. The fish were then allowed to acclimatize overnight. Starting the next day, behaviors were scored by a single observer, unaware of the strain identity of each fish, three times per day for 10 days (30 observation periods per individual fish). Observations of behavior were conducted at 0830 hours (one hour prior to the morning feeding), 1230 hours, and 1630 hours (one hour after the afternoon feeding) following methods developed in our laboratory [43,46]. Briefly, each fish was observed at the eye level of the observer from a distance of 0.5 m for a period of three seconds, and its vertical and horizontal location was recorded three times at one second intervals. Vertical location was coded as an integer representing the vertical zone occupied by the fish (1 = top, 6 = bottom); the final “Vertical Depth” score for each individual was computed as the mean across all 30 observation periods. The horizontal location of the fish was recorded as a binary variable, with 1 indicating the fish was within one body length of the front of the tank and 0 indicating the fish was not. “Horizontal Position” was calculated as mean horizontal location across all 30 observations and is an estimate of the proportion of time the animal spent within one body length of the front of the tank, nearest the human observer, over the course of the experiment. All behavior experiments were performed in a common rearing environment experimental design in which extreme care was taken to expose all fish to identical rearing conditions. These behaviors have been shown to have repeatabilities between 0.4 and 0.6 [46].

Sex of each fish was determined by inspection of external sex characteristics. Our goal was to test equal numbers of males and females from each strain; however, the final experiment was slightly imbalanced due to unequal sex ratios in the Nadia (male-biased) and SH (female-biased) strains. This did not affect our behavioral analyses, as we detected no differences in behavior between the sexes (data not shown).

All behavior data were analyzed using SAS, version 9.1 (SAS Institute, Cary, NC). We tested the effect of strain on behavior phenotypes using one-way Analysis of Variance (ANOVA) with the GLM procedure. The model included Sex, Domestication history, and Strain nested within Domestication history as fixed effects in the model. The residuals were visually examined to ensure that the assumption of normality was not violated.

### Tissue isolation and RNA extraction

After behavior assays were completed, fish were removed from the individual tanks and placed in one of sixteen 3-L group tanks. Four fish of the same sex and strain were assigned to each tank, resulting in four replicate tanks per strain. In the case of strains with biased sex ratios (see above), some tanks contained either 3 or 5 fish. The fish remained in these tanks during the period between collection of behavioral data and isolation of tissues for microarray analysis (one week).

On the sampling day, each fish was anesthetized in 170 mg L^−1^ tricaine methanesulfonate (MS222, Western Chemical Inc., Ferndale, WA), briefly blotted on a paper towel, and rapidly measured for standard length and body mass. The brain was then quickly removed and homogenized in TRIzol (Invitrogen, Carlsbad, CA). Brains from all individuals in a tank were homogenized together, for a total of two biological replicate pools per sex per strain (16 microarrays total). Total RNA was then extracted using the TRIzol method following manufacturer’s protocol. RNA quality was confirmed using gel electrophoresis and visual spectrophotometry using a Nanodrop spectrophotometer. All samples had 260/280 nm absorbance ratios between 1.85 and 2.05, and 260/230 nm absorbance ratios greater than 0.85.

### Microarray hybridization

For each array, 10 μg of total RNA were converted to cDNA. Biotinylated cRNA was then produced *in vitro* using the GeneChip expression 3′ amplification one-cycle target labeling kit (Affymetrix, Santa Clara, CA, USA). Affymetrix Zebrafish Genome Arrays (~14,900 transcripts) were hybridized with fragmented biotinylated cRNA for 16 h at 45 °C with constant rotation (45 rpm), and processed using the Affymetrix GeneChip Fluidic Station 450. Streptavidin-conjugated phycoerythrin (SAPE) was used for staining, followed by amplification using a biotinylated anti-streptavidin antibody. This was followed by another round of SAPE prior to scanning using a GeneChip Scanner 3000 (Affymetrix). All microarray procedures were performed at the Genomics Core Facility of the Center for Reproductive Biology at Washington State University (Pullman, WA).

### Statistical analysis of microarray data

CEL files containing raw data were then processed and analyzed using R software and Bioconductor packages [104,105]. These CEL files have been deposited with the NCBI Gene Expression Omnibus (GEO, Accession: GSE38729, http://www.ncbi.nlm.nih.gov/geo). Microarray hybridization data were examined for physical anomalies on the chip by pseudochip and residual error visualizations. Quality assurance of microarray data was completed using the affyQAReport function from the Bioconductor package affyQCReport. Hybridization and housekeeping controls, RNA degradation, sample clustering, NUSE plots, LPE plots, and RLE plots all showed high quality data (not shown) and no chips were removed. The arrays were then pre-processed using the Robust Multi-array Average (RMA) procedure [106-108] using the affy package [109]. Next, unexpressed and low variability genes were removed by unbiased filtering. Affymetrix present-marginal-absent (PMA) calls were determined using a *P*-value cut off for absent of greater than 0.04 and present less than 0.04; marginal calls were treated as absent. Unexpressed genes were then defined as having a signal less than the expression value at which 99 % of genes were called as absent across all samples. A filter on interquartile range was also applied to remove genes with low variability. Genes with an interquartile range of less than 0.5 across all chips in the experiment were excluded, reducing the dataset further to 7,958 genes.

Signal intensities were also examined at the probe level to identify single feature polymorphisms (SFPs), differences at the probe level due to genetic polymorphisms rather than expression differences, which may also impact computed expression values. Briefly, in R using previously described methods [61], the RMA normalized expression estimate for each probe set was subtracted from background corrected and normalized expression levels at individual probes within the probe set. Normalized residuals were analyzed using significance analysis of microarrays (SAM) [110] within the siggenes package to detect features with a significant effect for strain (FDR adjusted α < 0.01). A total of 3,199 genes with significant SFPs were then removed from the analysis.

The Linear Models for Microarray Data (limma) package was then used to perform differential expression analysis on the filtered gene list using a linear model on log_2_ signal values with an empirical Bayes correction to the variance [64,65]. Comparisons of interest were extracted through contrasts, and *P*-values were corrected for multiple comparisons using the Benjamini and Hochberg method (FDR = 5 %) [111]. The data were initially analyzed with a fully crossed factorial model of Strain and Sex. However, because the Sex and interaction terms were not significant for the vast majority of genes (see Results), we also tested for differential expression among strains using a model that excluded sex. The effect of domestication history was tested using contrasts between wild and domesticated strains.

Functional analyses of the resulting gene lists were performed using DAVID version 6.7 [67,68]. Functional categories were evaluated against the probability that they appeared in lists of differentially expressed genes at random based on their representation in the full list of genes that were classified as expressed in the zebrafish brain in our analysis. Functional classifications considered in the analysis included gene ontologies (GOs, [112]), COG ontologies, protein domains from PIR superfamilies, Interpro, and SMART databases, KEGG pathways, SP PIR keywords, and Up Seq features. Analysis of gene ontologies used the “FAT” option which filters the output, removing upper level GOs primarily from the top five levels of the hierarchy, in favor of more specific GOs that are often more informative. Categories were considered overrepresented if the EASE score was less than 0.05. We also report P-values adjusted for multiple tests using the Benjamini-Hochberg correction [111]. The Functional Annotation Clustering tool was used to identify categories with overlapping lists of differentially expressed genes.

### qRT-PCR validation

Technical validation of microarray results was performed using qRT-PCR on the sixteen sample pools used in the microarray analysis. Six genes were selected in order to represent multiple patterns of expression, including genes that differed among strains, between wild and domesticated strains, or between the sexes. The primer sequences for these genes are provided in Additional file 4. For each sample, 400 ng total RNA were converted to cDNA in a 20 μl total volume containing 100 ng random primers (Invitrogen, Carlsbad, CA), 0.5 mM dNTP, 0.05 M DTT, 1X SuperScript reaction buffer, and SuperScript II reverse transcriptase (100 U; Invitrogen). The resulting cDNA was diluted 1:5 with TE (pH 8) and used as template for qRT-PCR using SYBR®-Green PCR Master Mix (Applied Biosystems, Foster City, CA) on an ABI 7900HT Fast Real-Time PCR System, following the manufacturer’s protocol. Specificity of the qRT-PCR was verified through melting curve analysis. β actin was used as a reference gene because expression of this gene did not differ among strains in the microarray analysis. Standard curves and negative controls were included for both target and reference genes on every plate. Standard curves consisted of a 1:4 dilution series of a cDNA pool created from all 16 samples.

qRT-PCR data were analyzed using mixed model analysis of covariance (ANCOVA) using the *lm* function in R. This approach analyzed expression of the target gene (C_T_s) while simultaneously normalizing expression by including expression of the reference gene (C_T_s) as a covariate in the model [88,113]. We employed a fully crossed factorial model using the main effects of Strain and Sex. When the effect of Strain was significant, pairwise comparisons were performed using Tukey’s Studentized Range test.

## Competing interests

The authors have no competing interests.

## Authors’ contributions

BDR conceived and designed the experiment. BDR and RED drafted the manuscript. EJC reared fish and collected behavior data. EJC and RED prepared RNA samples for microarray analysis. RED, EJC, SB, and SMW performed the qRT-PCR validations. MLS, BDR and RED performed statistical analyses. All authors read and approved the final manuscript.

## Supplementary Material

Additional file 1 **Microarray analysis revealed 1,752 probe sets that differed significantly in expression among strains of zebrafish (*Danio rerio*).** Click here for file

Additional file 2 **Functional categories overrepresented among genes differentially expressed among strains of zebrafish (*Danio rerio*), revealed by DAVID analysis.** Click here for file

Additional file 3 Microarray analysis revealed 112 probe sets that differed significantly between domesticated and wild zebrafish strains. Click here for file

Additional file 4 Genes and primers for validation of microarray results with quantitative Real-Time PCR (qRT-PCR). Click here for file
